# Multiplex Gastrointestinal Panel Testing in Hospitalized Patients With Acute Diarrhea in Thailand

**DOI:** 10.1093/ofid/ofae322

**Published:** 2024-06-15

**Authors:** Anupop Jitmuang, Panuwat Lertlaksameewilai, Arnon Poorichitiporn, Navin Horthongkham, Methee Chayakulkeeree

**Affiliations:** Department of Medicine, Faculty of Medicine Siriraj Hospital, Mahidol University, Bangkok, Thailand; Department of Medicine, Rayong Hospital, Rayong, Thailand; Department of Medicine, Bhuddhachinaraj Hospital, Pitsanulok, Thailand; Department of Microbiology, Faculty of Medicine Siriraj Hospital, Mahidol University, Bangkok, Thailand; Department of Medicine, Faculty of Medicine Siriraj Hospital, Mahidol University, Bangkok, Thailand

**Keywords:** diarrhea, hospitalized patients, multiplex gastrointestinal panel test, traditional diagnoses, factors associated with test result

## Abstract

**Background:**

Multiplex gastrointestinal (GI) panel testing is widely used for outpatient diagnosis of diarrhea. However, the clinical practicality of multiplex testing in hospitalized diarrheal subjects has not yet been thoroughly elucidated.

**Methods:**

We enrolled hospitalized subjects with acute diarrhea. The subjects’ stool samples were collected in triplicate; 1 sample was tested using traditional diagnoses, and the other 2 were tested using Allplex (AP) and FilmArray (FA) GI panel testing. Clinical data were reviewed and analyzed.

**Results:**

Of the 199 subjects, 92 (46.5%) were male, and the mean age was 66.3 years. The median (interquartile range) onset of diarrhea was 6 (2­–14) days after hospitalization. One hundred fifty-one patients (75.9%) had sepsis, and 166 (83.4%) had received prior or were receiving current antimicrobial therapy. Positive stool cultures were obtained from 4/89 (4.5%), and *Clostridioides difficile* toxin gene tests were positive in 14/188 (7.4%) patients. AP and FA multiplex tests were positive for GI pathogens in 49/199 (24.6%) and 40/199 (20.1%), respectively. The target most frequently detected by AP was *Aeromonas* spp. Both assays commonly detected enteropathogenic *E. coli* (EPEC), *C. difficile* toxin gene, and *Salmonella* spp.; neither assay detected pathogens in 75.4% and 79.9%. Fever (odds ratio [OR], 2.05; 95% CI, 1.08–3.88; *P* = .028), watery diarrhea (OR, 2.69; 95% CI, 1.25–5.80; *P* = .011), and antimicrobial therapy (OR, 2.60; 95% CI, 1.18–5.71; *P* = .018) were independent factors associated with the negative multiplex test result.

**Conclusions:**

Multiplex GI panel testing effectively detects enteric pathogens associated with diarrhea in hospitalized subjects. The etiology remains undiagnosed in >75% of cases. Factors contributing to negative test results should be considered before implementing the tests.

Acute infectious diarrhea is one of the leading causes of infectious diseases worldwide associated with the ingestion of contaminated water or food [1]. This condition has several causative agents, such as toxins, bacteria, viruses, protozoa, and parasites [2]. Meanwhile, onset of acute diarrhea at hospitalization is associated with more diverse conditions. For example, it might be a delayed onset of community-acquired infectious diarrhea, or it might occur after hospitalization for a few days to weeks, called nosocomial diarrhea. Nosocomial diarrhea is a common problem in hospitalized patients, attributable to infectious etiologies, such as *Clostridioides difficile*, norovirus, and rotavirus [3], and noninfectious conditions, such as medications, chemotherapeutic agents, enteral feeding, medical illnesses, and malabsorption [3, 4]. Acute diarrhea in hospitalized patients is a medical complex that requires systematic clinical evaluation and laboratory diagnoses to identify the etiology.

Traditional laboratory diagnoses generally rely on a combination of microscopic examination, stool cultures, and immunological assays. However, these traditional methods are insensitive and time-consuming, and ∼60% of hospitalized diarrheal patients have not yet learned the specified etiologic agents [5]. In addition, *Campylobacter*, norovirus, adenovirus, and cryptosporidia are essential gastrointestinal (GI) pathogens among immunosuppressed individuals [3, 6]. These pathogens may be underrecognized and not tested routinely in hospitalized patients. A multiplex polymerase chain reaction (PCR)–based assay has been widely used to diagnose enteric pathogens, particularly in outpatient settings [7]. It can simultaneously identify several GI pathogens and exhibits higher sensitivity with faster turnaround times than traditional methods [7–9]. Therefore, the American College of Gastroenterology (ACG) recommends using multiplex molecular tests as an adjunct to traditional stool diagnostic testing [10].

Allplex (Seegene, Seoul, South Korea) and FilmArray (BioFire Diagnostics, Salt Lake City, UT, USA) gastrointestinal panel assays are the 2 commercial multiplex PCR–based GI panel tests available to diagnose diarrheal disease in Thailand in recent years. Diarrhea in hospitalized patients is a relatively more complicated condition than in outpatient cases, and multiplex PCR testing may assist in the early detection of the etiology of this condition. Our institute is the largest university-based hospital for tertiary care. Many patients experience increased bowel movements after being hospitalized, and traditional stool testing fails to provide a diagnosis. Moreover, few studies have examined the application of the multiplex GI panel test in inpatient clinical practice. Therefore, this study aims to evaluate the clinical practicality of the AP and FA assays when applied to stool diagnostic tests in hospitalized patients with acute diarrhea.

## METHODS

We conducted a cross-sectional observational study of adults aged ≥18 years admitted to the general medical wards at Siriraj Hospital, Bangkok, Thailand, from December 2016 to December 2018. We enrolled subjects who presented with a short passage of ≥3 loose or liquid stools per day [1] at the beginning of hospitalization. We excluded patients with chronic diarrhea, those who had symptoms for >30 days, and those who received laxative agents for medical treatment or bowel preparation. Subjects were assessed to confirm eligibility and provided written informed consent. After enrollment, the subjects’ stool specimens were collected in triplicate. Per the protocol of this pragmatic study, a set of fresh stool samples was sent to the microbiology laboratory for traditional laboratory diagnoses, per the request of the attending physician. The other 2 sets were fresh stool and Cary Blair–preserved stool samples stored at −20°C in the Molecular Diagnostic Unit, Department of Microbiology, for further testing using the AP and FA assays. In addition, patient demographic data, comorbidities, concurrent medical illness, stool characteristics, stool examination and findings, microbiological results, and clinical manifestation, onset, duration, and frequency of diarrhea were collected. The Scientific Ethics Committee approved this study, Siriraj Institutional Review Board (COA Si 795/2016 and Si 568/2023).

### Traditional Stool Laboratory Diagnoses

A set of stool samples was obtained from each patient's ward and immediately sent to the microbiology and parasitology laboratory for traditional tests. Traditional diagnoses comprised direct microscopic examination to detect white blood cells, red blood cells, ova and parasites (O&P), and stool cultures on blood, MacConkey, Hektoen, thiosulfate-citrate-bile salts-sucrose (TCBS), and modified semisolid Rappaport-Vassiliadis (MSRV) agars, including gram-negative broth for the detection of *Salmonella*, *Shigella*, *Aeromonas*, *Plesiomonas*, and *Vibrio*. All media were incubated at 35°C–37°C in ambient air and held for 3 days before being reported as negative. Stool cultures and isolations for *Campylobacter* spp. and *Yersinia enterocolitica* are not available in our institute. Conventional biochemical testing and automated phenotypic identification systems (Vitek-2; bioMérieux, Durham, NC, USA) were used to identify stool isolates. Additionally, the BD MAX Cdiff assay (BD Diagnostics, Heidelberg, Germany) was used for detection of the *C*. *difficile* toxin B gene.

### Multiplex PCR-Based Gastrointestinal Panel Testing

The 2 multiplex GI panel tests, the AP and FA assays, were used to test stool samples in parallel to compare diagnostic results between the 2 assays. The microbiology staff responsible for the multiplex GI panel test was blinded to the results of the traditional test. The AP assay can detect 25 gastrointestinal targets from fresh stool samples, as described elsewhere [11]. In summary, 100–200 mg of stool samples was harvested using a swab and suspended in NucliSENS lysis buffer (bioMerieux, Marcy-l’Etoile, France), followed by pulse vortexing and incubation at room temperature. The stool suspension obtained was centrifuged to generate the supernatant. The supernatant was applied for nucleic acid extraction using the automated NucliSENS easyMAG extraction system (bioMerieux, Marcy-l’Etoile, France). The nucleic acids of each target were processed and run using the Real-Time PCR Allplex system. According to the manufacturer's instructions, the specific GI target sequence was reported as a cycle threshold (Ct) value using Seegene Viewer analysis.

The FA assay can identify 22 GI targets from Cary Blair–preserved stool samples, as described elsewhere [12]. This study used the final Food and Drug Administration–cleared FA assay, in which the result of the *Aeromonas* target was not analyzed or reported. An individually packaged FA GI pouch was placed in the FilmArray pouch loading station. The sample injection vial and the hydration injection vial were placed in the pouch loading station and the hydration injection vial was inserted to drain the hydration solution into the pouch. A transfer pipette was used to draw the stool sample to be mixed with the buffer solution in the sample injection vial. The sample mix was then loaded into the pouch, which in turn was loaded into the FilmArray instrument (the FilmArray 2.0 system), where automated nucleic acid extraction, multiplex PCR, and postamplification analysis were performed. FilmArray software performed a high-resolution DNA melting analysis on the PCR products. It measured the fluorescence signal generated in each well before reporting the result of each target according to the manufacturer's instructions.

Clinical evaluation and patient management were performed by the attending physician when there were discordant results between tests. The study investigators were not involved in clinical management.

### Statistical Analysis

Continuous data are presented as mean ± SD for normally distributed data and median and interquartile range (IQR) for non–normally distributed data. Categorical data are presented as frequency and percentage. The chi-square or Fisher exact test was used to compare categorical variables between groups. In addition, an independent *t* test was used to compare normally distributed continuous data, and the Mann-Whitney *U* test was used to compare non–normally distributed continuous data. Multivariate analysis through the multiple logistic regression model (backward stepwise) included all variables with a *P* value <.2 from univariate analysis. All statistical analyses were performed using SPSS Statistics (version 18.0) software (SPSS, Inc., Chicago, IL, USA), and a 2-sided *P* value <.05 was considered statistically significant. In addition, agreement between traditional stool diagnoses and the 2 multiplex GI panel tests was evaluated using Cohen's kappa (κ) coefficient. Agreement was classified as very good (κ = 0.81–1.00), good (κ = 0.61–0.80), moderate (κ = 0.41–0.60), fair (κ = 0.21–0.40), or poor (κ = 0.00–0.20).

## RESULTS

### Baseline Characteristics, Clinical Manifestations, and Results of Traditional Laboratory Diagnoses

The study enrolled 199 subjects with acute diarrhea at hospital onset; 92 (46.5%) were male, and the mean age (SD) was 66.3 (17.8) years. Diabetes mellitus (40.4%) was a common comorbidity. Among eligible subjects, 45 (22.6%) received immunosuppressive therapy, 56 (28.1%) had malignancies, and 14 (7.1%) had neutropenia (Table 1). More than half of the subjects (55.3%) suffered from severe noninfectious medical illnesses as a cause of hospitalization. The median onset of diarrhea during hospital stay (IQR) was 6 (2–14) days, the mean frequency of diarrhea was 5.8 (±2.0) times/day, and the median duration of diarrhea (IQR) was 7 (4–12) days. Most subjects had watery diarrhea (81.9%) and fever (61.8%) at onset, while only 18.1% had mucous or bloody diarrhea. Attending physicians requested stool sample collection a median (IQR) of 4 (2–7) days after the onset of diarrhea. One hundred fifty-one (75.9%) subjects had a diagnosis of sepsis at the time of onset; pneumonia (39.2%), urinary tract infection (25.6%), and intra-abdominal infection (11.6%) were the leading causes of sepsis. Furthermore, most of the subjects (83.4%) had received prior or were receiving concurrent antimicrobial therapy.

**Table 1.** Baseline Characteristics, Clinical Manifestations, and Results of Traditional Laboratory Diagnoses

Characteristics	Total
Male sex, No. (%)	92 (46.5)
Mean age (SD), y	66.3 (17.8)
Comorbid condition, No. (%)	
** **Diabetes mellitus	80 (40.4)
Chronic kidney disease	59 (29.8)
Malignancy	56 (28.1)
Receiving immunosuppressive therapy	45 (22.6)
Autoimmune disease	19 (9.6)
Neutropenia	14 (7.1)
Others	22 (11.1)
Cause of hospitalization,^a^ No. (%)	
Infection-related	89 (44.7)
Non-infection-related	110 (55.3)
Clinical manifestation at the diarrheal onset	
Time from admission to onset, median (IQR), d	6 (2–14)
Frequency, mean (SD), times/d	5.8 (2.0)
Duration,^b^ median (IQR), d	7 (4–12)
Fever, No. (%)	123 (61.8)
Vomiting, No. (%)	33 (16.6)
Abdominal pain, No. (%)	30 (15.2)
Watery diarrhea, No. (%)	163 (81.9)
Mucus or bloody diarrhea, No. (%)	36 (18.1)
Time to stool sampling,^c^ median (IQR), d	4 (2–7)
Concurrent infection at onset, No. (%)	
Sepsis from any causes	151 (75.9)
Pneumonia	78 (39.2)
Urinary tract infection	51 (25.6)
Intra-abdominal infection	23 (11.6)
Skin and soft tissue infections	21 (10.6)
Primary bacteremia	11 (5.5)
Others	16 (8.0)
Unclear infectious foci	7 (3.5)
Prior or current antimicrobial therapy, No. (%)	166 (83.4)
Hematologic findings	
Hemoglobin, mean (SD), g/dL	10.4 (5.1)
WBC counts, median (IQR), cells/mm^3^	9980 (6480–13 420)
Results of stool microscopic examination (n = 193)	
No WBCs or RBCs	173 (89.6)
Positive WBCs and/or RBCs	20 (10.4)
No. of stool WBCs	
** **1–3 cells/HPF	11 (55.0)
>3–5 cells/HPF	2 (10.0)
>5–10 cells/HPF	2 (10.0)
>10–30 cells/HPF	3 (15.0)
>30 cells/HPF	1 (5.0)
No. of stool RBCs	
1–5 cells/HPF	4 (20.0)
>5–10 cells/HPF	3 (15.0)
>10 cells/HPF	2 (10.0)
Results of traditional stool diagnostic tests, No. (%)	
*Clostridium difficile* toxin gene detection positive	14/188 (7.4)
Stool cultures positive	4/89 (4.5)^d^
Stool O&P positive	1/65 (1.5)^e^

Abbreviations: HPF, high-power field; IQR, interquartile range; O&P, ova and parasite; RBCs, red blood cells; WBCs, white blood cells.

^a^Infection-related causes of hospitalization, including severe sepsis, pneumonia, urinary tract infection, hepatobiliary infection, skin and soft tissue infection, bloodstream infection, etc. Non-infection-related causes of hospitalization included cerebrovascular disease, coronary artery disease, heart failure, exacerbation of airway diseases and respiratory failure, acute renal failure, severe metabolic disturbance, hematologic disorders, etc.

^b^The total number of days from the onset of diarrhea to the time of diarrhea resolved.

^c^The total number of days from the onset of diarrhea to the time of study enrollment.

^d^Stool cultures positive for *Salmonella* non-Typhi group B (1), *Salmonella* non-Typhi group C (1), *Aeromonas veronii* (1), and dual *Salmonella* non-Typhi group C and *A*. *veronii* (1).

^e^ *Strongyloides stercoralis* detected.

Traditional laboratory findings are shown in Table 1. Of 199 subjects, 193 had fresh stool samples for microscopic examination, but only 20 (10.4%) had positive findings for fresh stool white blood cells (WBCs) and/or red blood cells (RBCs). Stool samples from 6 subjects for the microscopic exam were unavailable as the physician considered canceling the test. A total of 188 subjects provided stool samples for traditional *C. difficile* toxin gene detection. Additionally, 89 provided stool cultures, and 65 underwent O&P examinations. The results showed that only 14 subjects (7.4%) tested positive for *C. difficile* toxin gene, while a small proportion of subjects tested positive for stool cultures (4.5%) and O&P (1.5%) (Table 1).

### Identification of Gastrointestinal Pathogens by Multiplex PCR Panels Among 199 Hospitalized Patients

The results of testing enteric targets by the AP and FA panels were found to be similar, as shown in Table 2. The most frequently detected target by the AP was *Aeromonas* spp., which is not a target for the FA. However, both assays commonly detected enteropathogenic *E. coli* (EPEC), *C. difficile* toxin gene, and *Salmonella* spp. Although there was a slight difference in EPEC between FA and AP, this difference was insignificant (*P* = .163). Overall, the AP and FA assays detected 70 (35.2%) and 64 (32.2%) targets, respectively. In addition, the AP and FA assays detected enteric targets in stool samples from 49 (24.6%) and 40 (20.1%) subjects, respectively. Among the subjects with positive GI panels, the AP assay detected that 37 (18.6%) had a single target, 8 (4.0%) had dual targets, and 4 (2.0%) had 3 targets. Meanwhile, the FA assay showed that 26 (13.1%) had a single target, 7 (3.5%) had dual targets, and 7 (3.5%) had ≥3 positive targets. Out of 199 subjects, 150 (75.4%) and 159 (79.9%) had negative test results by the AP and FA assays, respectively. There was no statistical difference between the numbers of target detection using the AP and FA assays (*P* = .381).

**Table 2.** Identification of Gastrointestinal Pathogens by Multiplex PCR Panels Among 199 Hospitalized Patients

Enteric Pathogen	AP Panel (n = 199)		FA Panel (n = 199)		*P* Value
	No. Positive	(%)	No. Positive	(%)	
Bacteria					
* Aeromonas* spp.	23	11.6	na		-
* Campylobacter* spp.	1	0.5	4	2.0	.372
* C. difficile* toxin gene	13	6.5	13	6.5	1.00
* C. difficile* (hypervirulent)	0		na		-
* *Enteroaggregative *E. coli*	4	2.0	6	3.0	.522
* *Enteropathogenic *E. coli*	10	5.0	17	8.5	.163
* *Enterotoxigenic *E. coli*	1	0.5	1	0.5	1.00
* E. coli* O157	4	2.0	1	0.5	.372
* Plesiomonas shigelloides*	na		3	1.5	-
* *Shiga toxin–producing *E. coli*	1	0.5	1	0.5	1.00
* Salmonella* spp.	7	3.5	8	4.0	.792
* Shigella* spp./enteroinvasive *E. coli*	0		0		na
* Vibrio* spp.	1	0.5	3	1.5	.623
* V. cholerae*	na		0		-
* Yersinia enterocolitica*	0		0		na
Viruses					
* *Adenovirus F serotypes 40/41	0		0		na
* *Astrovirus	0		0		na
* *Norovirus G1/G2	4	2.0	3	1.5	.703
* *Rotavirus A	1	0.5	1	0.5	1.00
* *Sapovirus	0		2	1.0	.499
Parasites					
* Blastocystis hominis*	0		na		-
* Cryptosporidium* spp.	0		0		na
* Cyclospora cayetanensis*	0		0		na
* Dientamoeba fragilis*	0		na		-
* Entamoeba histolytica*	0		0		na
* Giardia lamblia*	0		1	0.5	1.00
* *Total targets identified	70	35.2	64	32.2	.525
* *No. of patients with positive test	49	24.6	40	20.1	.279
* *1 target positive	37	18.6	26	13.1	.381^a^
* *2 targets positive	8	4.0	7	3.5	
* *≥3 targets positive	4	2.0	7	3.5	
* *Negative tests	150	75.4	159	79.9	

Abbreviations: AP, Allplex; FA, FilmArray; na, not applicable.

^a^A comparison of 1, 2, and ≥3 positive vs negative tests between AP and FA assays revealed a *P* value of .381.

### Agreement Between Different Stool Diagnostic Methods

The detection of *C. difficile* toxin genes in stool samples when tested by the conventional PCR assay (BD MAX Cdiff assay, BD Diagnostics, Heidelberg, Germany) and the 2 multiplex GI panel assays exhibited substantially good agreement (κ = 0.835; 95% CI, 0.676–0.993), as shown in Table 3A. The molecular assays also detected *Salmonella* targets, relatively consistent with standard cultures. The detection of *Salmonella* spp. tested by the standard cultures and the 2 multiplex GI panel assays exhibited good agreement (κ = 0.738; 95% CI, 0.390–1.00) (Table 3B). The study found that the AP was able to detect 2 *Aeromonas* targets, but the agreement with the standard culture method for detecting *Aeromonas* was only fair (κ = 0.339; 95% CI, −0.013 to 0.692), as shown in Table 3C. Commonly detected enteric targets, such as *C. difficile* toxin genes, EPEC, *Salmonella* spp., norovirus, and EAEC, were analyzed to study the agreement between the 2 multiplex GI panel tests (Table 3D). We did not analyze the agreement of the *Aeromonas* target as it is an off-target for FA testing. The AP and FA assays demonstrated excellent agreement in detecting the *C. difficile* toxin genes (κ = 0.918; 95% CI, 0.805–1.00) and *Salmonella* (κ = 0.931; 95% CI, 0.796–1.00) and good agreement for the EAEC (κ = 0.795; 95% CI, 0.518–1.00) targets. Meanwhile, the agreement of the 2 assays was not promising in detecting the norovirus (κ = 0.662; 95% CI, 0.221–1.00) or EPEC (κ = 0.360; 95% CI, 0.102–0.619) targets (Table 3).

**Table 3.** An Agreement Between Different Stool Diagnostic Methods

A, Agreement between traditional and 2 multiplex GI panel testing for the detection of *C. difficile* toxin gene (n = 188)						
*C. diffcile* PCR assay^a^	The AP GI Panel		κ (95% CI)	The FA GI Panel		κ (95% CI)
	Pos	Neg		Pos	Neg	
Pos	11	3	0.835 (0.676–0.993)	11	3	0.835 (0.676–0.993)
Neg	1	173		1	173	

B, Agreement between traditional stool cultures and 2 multiplex GI panel tests for the detection of *Salmonella* spp. (n = 89)						
Stool cultures	The AP GI Panel		κ (95% CI)	The FA GI Panel		κ (95% CI)
	Pos	Neg		Pos	Neg	
Pos	3	1	0.738 (0.390–1.00)	3	1	0.738 (0.390–1.00)
Neg	1	84		1	84	

C, Agreement between traditional stool cultures and the AP assay for the detection of *Aeromonas* spp. (n = 89)						
Stool cultures	The AP GI Panel		κ (95% CI)			
	Pos	Neg				
Pos	2	0	0.339 (−0.013 to 0.692)			
Neg	7	80				

D, Agreement between the 2 multiplex GI panel tests for detection of common enteric targets^b^ (n = 199)				
*C. difficile* toxin gene		The FA GI Panel		κ (95% CI)
	The AP GI Panel	Pos	Neg	
	Pos	12	1	0.918 (0.805–1.00)
	Neg	1	185	

Enteropathogenic *E. coli*		The FA GI Panel		
	The AP GI Panel	Pos	Neg	
	Pos	4	0	0.360
	Neg	13	182	(0.102–0.619)

*Salmonella*		The FA GI Panel		
	The AP GI Panel	Pos	Neg	
	Pos	7	0	0.931
	Neg	1	191	(0.796–1.00)

Norovirus		The FA GI Panel		
	The AP GI Panel	Pos	Neg	
	Pos	2	1	0.662
	Neg	1	195	(0.221–1.00)

Enteroaggregative *E. coli*		The FA GI Panel		
	The AP GI Panel	Pos	Neg	
	Pos	4	0	0.795
	Neg	2	193	(0.518–1.00)

Abbreviations: AP, Allplex; FA, FilmArray; GI, gastrointestinal; κ, Cohen's kappa coefficient; Neg, negative; PCR, polymerase chain reaction; POS, positive.

^a^ *C. difficile* toxin B gene testing (n = 188) using the BD MAX Cdiff assay (BD Diagnostics, Heidelberg, Germany).

^b^Excluding *Aeromonas* spp. as the off-target for FA testing.

### Factors Associated With Negative Test Results for the Multiplex GI Panel Assays

This study used 2 multiplex GI panel tests to identify enteric pathogens in stool samples. The tests yielded positive results in only 24.6% using the AP assay and 20.1% using the FA assay. However, a significant number of participants (75.4%) tested negative for enteric pathogens using the AP assay, while 79.9% tested negative using the FA assay. We need to know which clinical factors could affect the overall results of the multiplex GI panel testing. The study populations were categorized into 2 groups: 1 with a positive test result for either AP or FP (60) and another with negative test results for both AP and FP (139), as shown in Table 4. Several factors contributed to a significant difference between subjects, with either the AP or FP testing positive and subjects with both panels testing negative, such as age, diagnosis of sepsis at onset, preceding or concurrent antimicrobial therapy, the interval between admission and diarrheal onset, having fever, watery-type diarrhea, percentage of peripheral monocytes, and percentage of peripheral eosinophils. From Table 5, the univariate analysis demonstrates that several factors had a significant association with negative test results for both AP and FP, such as age (odd ratio [OR], 1.02; 95% CI, 1.00–1.04; *P* = .030), fever (OR, 2.02; 95% CI, 1.09–3.75; *P* = .025), watery-type diarrhea (OR, 2.50; 95% CI, 1.20–5.24; *P* = .016), days from admission to onset (OR, 1.03; 95% CI, 1.00–1.06; *P* = .029), preceding or concurrent antimicrobial therapy (OR, 2.61; 95% CI, 1.22–5.60; *P* = .014), and sepsis at onset (OR, 1.98; 95% CI, 1.01–3.91; *P* = .048). However, multivariate analysis revealed that fever (OR, 2.05; 95% CI, 1.08–3.88; *P* = .028), watery diarrhea (OR, 2.69; 95% CI, 1.25–5.80; *P* = .011), and preceding or concurrent antimicrobial therapy (OR, 2.60; 95% CI, 1.18–5.71; *P* = .018) were independent factors strongly associated with a negative multiplex GI panel.

**Table 4.** Factors Associated With the Results of Multiplex GI Panel Testing

Factors	Overall Results		*P* Value
	Either AP or FP Test Positive (n = 60)	Both AP and FP Tests Negative (n = 139)	
Median age (IQR), y	62.0 (53.5–76.5)	71.0 (58.00–81.00)	.020
Gender, No. (%)			
Male	31 (51.7)	61 (43.9)	.312
Female	29 (48.3)	78 (56.1)	
Comorbid condition, No. (%)			
Diabetes mellitus	25 (41.7)	55 (39.6)	.782
Immunosuppressive therapy	14 (28.6)	31 (20.6)	.250
Chronic kidney disease	14 (23.3)	45 (32.4)	.200
Hematologic malignancy	10 (16.7)	20 (14.4)	.680
Solid malignancy	6 (10.0)	20 (14.4)	.399
Autoimmune disease	5 (8.3)	14 (10.1)	.702
Neutropenia	4 (6.7)	10 (7.2)	1.000
Cause of hospitalization, No. (%)			
Infection-related	26 (43.3)	63 (45.3)	.796
Non-infection-related	34 (56.7)	76 (52.5)	
Sepsis at onset, No. (%)	40 (66.7)	111 (79.9)	.046
Prior or current antimicrobial therapy, No. (%)	44 (73.3)	122 (87.8)	.012
Median time from admission to diarrheal onset (IQR), d	5.0 (1.0–8.5)	6.0 (2.0–15.0)	.035
Median frequency of diarrhea (IQR), times	5.0 (4.0–6.0)	6.0 (5.0–7.0)	.353
Median duration of diarrhea^a^ (IQR), d	7.0 (4.0–12.0)	7.0 (4.0–12.0)	.896
Clinical manifestation at onset, No. (%)			
Fever	30 (50.0)	93 (66.9)	.024
Vomiting	12 (20.0)	21 (15.1)	.394
Abdominal pain	11 (18.3)	19 (13.7)	.399
Type of diarrhea			
Watery type	43 (71.7)	120 (86.3)	.014
Mucous or bloody type	17 (28.3)	19 (13.7)	
Median time to stool sampling^b^ (IQR), d	3.00 (2.00–6.50)	4.00 (2.00–7.00)	.896
Hematologic findings			
Median hemoglobin (IQR), g/dL	9.30 (7.90–11.20)	9.00 (8.20–10.20)	.347
Median WBC count (IQR), cells/mm^3^	10 760 (5360–14 300)	9605 (6570–12 925)	.444
Mean neutrophils (SD), %	73.64 (15.53)	71.48 (19.12)	.447
Median lymphocytes (IQR), %	12.05 (8.00–20.00)	13.50 (7.60–21.00)	.561
Median monocytes (IQR), %	6.90 (4.30–8.70)	5.40 (4.10–6.90)	.023
Median eosinophils (IQR), %	1.00 (0.10–3.00)	1.90 (0.50–3.30)	.045

Abbreviations: AP, Allplex; FA, FilmArray; GI, gastrointestinal; IQR, interquartile range; WBC, white blood cells.

^a^The total number of days from the onset of diarrhea to the time diarrhea resolved.

^b^The total number of days from the onset of diarrhea to the time of study enrollment.

**Table 5.** Univariate and Multivariate Logistic Regression Analysis of Factors Associated With the Negative Test Results for the Multiplex GI Panel Assays

Factors	Univariate Logistic Regression Analysis	*P* Value	Multivariate Logistic Regression Analysis	*P* Value
	Odd Ratio (95% CI)		Odd Ratio (95% CI)	
Age	1.02 (1.00–1.04)	.030	-	-
Fever at onset	2.02 (1.09–3.75)	.025	2.05 (1.08–3.88)	.028
Watery-type diarrhea	2.50 (1.20–5.24)	.016	2.69 (1.25–5.80)	.011
Time from admission to onset	1.03 (1.00–1.06)	.029	-	-
Prior or current antimicrobial therapy	2.61 (1.22–5.60)	.014	2.60 (1.18–5.71)	.018
Sepsis at onset	1.98 (1.01–3.91)	.048	-	-

Abbreviation: GI, gastrointestinal.

## DISCUSSION

Traditional stool diagnoses only identified the cause of diarrhea in a small number of hospitalized patients in this study. In contrast, when applied to diagnosing diarrhea in clinical practice, the AP and the FP assays were able to more substantially detect enteric targets in 24.6% and 20.1% of the subjects, respectively. Using multiplex GI panel assays enabled us to discover several enteric pathogens that are usually underdiagnosed by traditional tests; for example, diarrheagenic *E. coli* were more often identified. Diarrheagenic *E. coli*, especially EAEC and EPEC, which have not been routinely tested in a general microbiology laboratory, are highly prevalent among Thai patients hospitalized with acute diarrhea [13]. Therefore, these agents could be associated with diarrheal symptoms and need more attention in hospitalized subjects. The traditional tests used in this study provided poor yield to detect enteric pathogens among hospitalized patients with diarrhea. Several studies have demonstrated that traditional methods can identify enteric pathogens that cause diarrhea in a range of 8.3% to 27.7% [14–17]. The diagnostic results of traditional methods vary depending on the availability of stool testing methods in an individual facility. Due to resource limitations, traditional stool testing in our hospital has not been able to detect all enteric pathogens. In addition, the attending physicians only considered and selected traditional testing methods; in this study, we found that the selected stool test can limit the diagnostic yield to detect the etiology of diarrhea. Identifying the etiologic agents that cause diarrhea is usually not feasible based on clinical manifestations. Therefore, a laboratory testing panel with multiple pathogen detection is warranted. Several studies have included conventional methods, such as traditional and particular cultures, immunoassay, molecular testing, and electron microscopy, for stool diagnosis. However, those diagnostic methods detected the causative pathogens in only 17.8%–27.7% of cases [14, 16, 18]. Therefore, the sensitivity of traditional methods still needs to be promising for diagnosing the etiology.

In clinical practice, when applied to stool diagnostics, the multiplex GI panel test can detect enteric targets in 24.6% and 20.1% of hospitalized subjects using AP and FP, respectively. Similarly, Alejo-Cancho et al. demonstrated that multiplex GI panel testing provided positive stool sample results of 26% when deployed as an additional test among immunosuppressed hematologic patients with acute diarrhea [18]. Furthermore, Axelrad et al. showed that using GI panel testing provided significantly more positive results (29.2%) than using conventional methods (4.1%) in both inpatient and outpatient diarrhea [19]. Multiplex testing can increase the detection rate from 26%–29% to 54%–66% when used in community-onset diarrhea, particularly in pediatric or HIV-infected patients [16, 17, 20, 21]. However, the current study applied multiplex testing to diagnose diarrhea in hospitalized patients with distinct clinical characteristics, such as multiple comorbidities, receiving immunosuppressive therapy, delayed onset of diarrhea, and concurrent sepsis requiring antimicrobial therapy at the beginning. Diarrhea that occurs during hospitalization, as in our study, is attributable to more diverse infectious and noninfectious causes, such as *Klebsiella oxytoca*, cytomegalovirus, severe sepsis, medications, feeding-associated, lactose intolerance, and intestinal ischemia [3, 22]. Diagnosis of diarrhea, particularly in hospitalized patients, is not straightforward and should exclude various other causes, which can limit the diagnostic yield of multiplex panel tests.

According to our study, multiplex tests, apart from the traditional methods, could identify several enteric pathogens usually underdiagnosed in hospitalized patients with diarrhea, such as *Aeromonas* spp., diarrheagenic *E. coli*, *Vibrio* spp., *P*. *shigelloides*, *Campylobacter* spp., norovirus, rotavirus, sapovirus, and *G*. *lamblia*. Several studies elucidated that multiplex testing can significantly increase the proportions of stool samples positive for *Aeromonas* spp. [9], diarrheagenic *E. coli* (EHEC, EPEC, ETEC, EAEC, and STEC) [16, 19, 21], *Campylobacter* spp. [9, 16, 21], enteric viruses (norovirus, astrovirus, and sapovirus) [17, 19, 21], and protozoa (*G*. *lamblia*, *D. fragilis*, and *B. hominis*) [19, 21, 23]. Therefore, multiplex GI panel testing has been used as the standard diagnostic method to replace traditional diagnoses in some facilities; it is less labor-intensive and provides a higher positivity rate [9, 19]. Consistent with our studies, we found that testing with 2 different multiplex panels provided concordant results for the same enteric targets [13, 22, 23]. Surprisingly, the multiplex testing did not detect *D. fragilis* or *B. hominis* in stool samples in this study. These protozoa are frequently reported as stool carriage. *D. fragilis* has rarely been reported, and its prevalence in Thailand is unknown [24]. *D. fragilis* is often ignored, and its pathogenic potential is still unclear [25], which can result in the underreporting of this protozoan in our country. Meanwhile, the prevalence of *B. hominis* in Thailand is mainly associated with poor environmental hygiene, young age groups, rural areas, and exposure to livestock or domestic pets [26–28]. The local epidemiology, urbanization, sanitation, and patient settings of our study may differ from previous studies, which could have an impact on the diagnostic outcomes of the multiplex testing. Local epidemiological data and the type of enteric targets available in panel testing should be considered before changing the diagnostic workflow from traditional methods to multiplex PCR–based assays. For example, *Aeromonas*, one of the leading causative agents in Thailand [13], can be underdiagnosed using FA alone; conventional methods such as backup testing are still required, particularly when off-target organisms are suspected.

Most of the stool samples tested by multiplex testing discovered a single pathogen (Table 2), but this positive result should be carefully interpreted. The detected corresponding target may not represent actual infection; it might be a nonviable organism, colonization, or asymptomatic GI shedding [13]. Surprisingly, a nationwide study showed that 87.8% of healthy Thai individuals had asymptomatic enteropathogen carriage detected by qPCR [13]. Polage et al. found that >80% of high-risk hospitalized patients, such as those who have received antimicrobial exposure, intensive care, postchemotherapeutics, and organ transplants, suffer from nosocomial diarrhea caused by noninfectious causes, particularly from medications. Only 10%–20% of these patients have diarrhea associated with *C. difficile* [3]. In cases where the *C. difficile* PCR tests came out positive, around 55% of them had negative toxin immunoassay tests [29]. These cases had outcomes comparable to those without *C. difficile* detected. Our study found that using the multiplex panels resulted in a higher positive *C. difficile* toxin gene detection rate. However, the prevalence of toxigenic *C. difficile* isolates at our institute was only 9.2% [30]. It can be difficult to distinguish between patients with *C. difficile* infection and those who carry *C. difficile* colonization as positive PCR tests are insufficient to make a conclusive diagnosis. Therefore, the results of multiplex GI panel testing may be complicated by asymptomatic carriage. Symptoms, patient setting, other noninfectious causes, and local data must be considered before interpreting the test result. In addition, the AP and FA assays were able to detect multiple targets in the study populations. The AP assay showed dual detection in 4.0% of cases and detection of ≥3 targets in 2.0% of cases, while the FA assay showed dual detection in 3.5% of cases and detection of ≥3 targets in 3.5% of cases (as shown in Table 2). Although multiple target detection is not uncommon when using these assays, the rates vary and range from 15% to 31.5% [15–17, 20, 31]. The type of enteric pathogen co-detected dramatically differs according to study, geographic region, and study population. The clinical significance of pathogen co-detection remains unclear, and many enteric pathogens can reside and sometimes escape the GI tract without causing symptoms for several weeks [31]. Thus, mixed pathogen co-detection may limit utilization of multiplex GI panel testing as it is difficult to interpret and the clinical importance is still unknown.

More than 75% of the cases in the current study had negative multiplex GI panel tests. Several factors, such as aging, patient symptoms, diarrheal onset, sepsis, and treatment, had significantly different multiplex testing results, positive and negative. However, fever, watery diarrhea, and antimicrobial therapy were critical factors associated with negative test results (Table 5). Fever and concurrent sepsis requiring antimicrobial treatment at the beginning could dysregulate GI motility and absorption and alter the intestinal microbiota, resulting in watery-type diarrhea in subjects with negative test results [32, 33]. Thus, the clinician should be aware that stool samples from hospitalized patients with those critical factors may result in a negative multiplex test result. Careful case selection for multiplex PCR–based testing is needed to conserve hospital costs and resources, particularly in a resource-limited facility.

There are several limitations to the current study. Due to the pragmatic approach, the types of traditional stool diagnoses, including microscopic examination, bacterial cultures, and *C. difficile* toxin gene detection, were chosen mainly by the attending physicians. Those tests had limited diagnostic yields and were performed on only some study subjects. Only 4 of 89 cases had positive stool cultures. Therefore, more culture-positive samples are needed to compare the agreement of all enteric targets detected by multiplex PCR–based assays and traditional methods. A limited number of traditional methods used and the financial limitations of the study prevent us from performing a confirmatory and validation analysis of the all-discrepant results between the critical enteric pathogens, such as *Aeromonas*, *E. coli*, *Salmonella*, *P. shigelloides*, *Campylobacter* spp., and *Vibrio* spp., detected by multiplex testing and traditional methods. Furthermore, our study did not determine a causal relationship between the detected pathogens and the patients’ clinical findings, treatment, and outcomes, in which the PCR testing may not clearly distinguish actual infection from asymptomatic shedding, nonviable organisms, and false positivity. Also, clinical management based on the test results was not fully evaluated as they were not included in the primary outcomes. Thus, it is unclear whether the GI panel test results influence patient management and outcomes in this study. More than 75% of diarrheal cases in the study had negative multiplex test results, but other causes remained unclear and were not thoroughly evaluated. Other potential factors that cause diarrhea in subjects with negative multiplex test results will be an important area of future research. In addition, more cost-effectiveness investigations are needed.

## CONCLUSIONS

Multiplex GI panel testing in hospitalized patients detects more enteric pathogens associated with diarrhea than traditional methods. However, the positive test should be carefully interpreted based on local epidemiology and the patient setting. Finally, several critical factors associated with a negative test result, such as fever, watery diarrhea, and antimicrobial therapy, should prompt the clinician to search for other potential causes.
