# Correction to: Multiplex Gastrointestinal Panel Testing in Hospitalized Patients With Acute Diarrhea in Thailand

**DOI:** 10.1093/ofid/ofaf229

**Published:** 2025-04-18

This is a correction to: Anupop Jitmuang, Panuwat Lertlaksameewilai, Arnon Poorichitiporn, Navin Horthongkham, Methee Chayakulkeeree, Multiplex Gastrointestinal Panel Testing in Hospitalized Patients With Acute Diarrhea in Thailand, *Open Forum Infectious Diseases*, Volume 11, Issue 7, July 2024, ofae322, https://doi.org/10.1093/ofid/ofae322

The following changes have been made to the originally published manuscript.

In the section **METHODS**, the last sentence of the first paragraph has been changed to read: “The Scientific Ethics Committee approved this study, Siriraj Institutional Review Board (COA Si 795/2016 and Si 568/2023).” instead of “The Scientific Ethics Committee approved this study, Siriraj Institutional Review Board (COA Si 795/2016).”

